# Augmenter of Liver Regeneration Protects Renal Tubular Epithelial Cells From Ischemia-Reperfusion Injury by Promoting PINK1/Parkin-Mediated Mitophagy

**DOI:** 10.3389/fphys.2020.00178

**Published:** 2020-03-13

**Authors:** Dong-Ju Zhu, Xiao-Hui Liao, Wen-Qi Huang, Hang Sun, Ling Zhang, Qi Liu

**Affiliations:** ^1^Department of Nephrology, The Second Affiliated Hospital, Chongqing Medical University, Chongqing, China; ^2^Department of Nephrology, The Affiliated Hospital, Panzhihua University, Panzhihua, China; ^3^Key Laboratory of Molecular Biology for Infectious Diseases (Ministry of Education), Institute for Viral Hepatitis, Department of Infectious Diseases, The Second Affiliated Hospital, Chongqing Medical University, Chongqing, China; ^4^Department of Intensive Care Medicine, The Second Affiliated Hospital of Chongqing Medical University, Chongqing, China

**Keywords:** augmenter of liver regeneration, mitochondria, mitophagy, PINK1, Parkin, ischemia–reperfusion injury

## Abstract

Ischemia–reperfusion (I/R) is the most common cause of acute kidney injury (AKI) and can induce apoptosis in renal epithelial tubule cells. Mitochondrial dysfunction is one of the main reasons for I/R-induced apoptosis. Accumulating evidence suggests that PINK1/Parkin-mediated mitophagy possibly plays a renoprotective role in kidney disease by removing impaired mitochondria and preserving a healthy population of mitochondria. Our previous study showed that augmenter of liver regeneration (ALR) alleviates tubular epithelial cells apoptosis in rats with AKI, although the specific mechanism remains unclear. In this study, we investigated the role of ALR in I/R-induced mitochondrial pathway of apoptosis. We knocked down ALR with short hairpin RNA lentiviral and established an I/R model in human kidney proximal tubular (HK-2) cells *in vitro*. We observed that the knockdown of ALR aggravated mitochondrial dysfunction and increased the mitochondrial reactive oxygen species (ROS) levels, leading to an increase in cell apoptosis via inhibition of mitophagy. We also found that the PINK1/Parkin pathway was activated by I/R via confocal microscopy and Western blot. Furthermore, the knockdown of ALR suppressed the activation of PINK1 and Parkin. These findings collectively indicate that ALR may protect HK-2 cells from I/R injury by promoting mitophagy, and the mechanism by which ALR regulates mitophagy seems to be related to PINK1 and Parkin. Consequently, ALR may be used as a potential therapeutic agent for AKI in the future.

## Introduction

Acute kidney injury (AKI) is a common and severe renal disease characterized by a rapid loss of kidney function. Mounting studies reported that AKI is an independent risk factor for end-stage kidney disease and chronic kidney disease and that severe AKI can induce multiorgan dysfunction (Chawla et al., 2014; Ferenbach and Bonventre, 2015; Venkatachalam et al., 2015). No effective treatment is available; thus, severe AKI is accompanied with a high mortality and has become a global public health issue and socioeconomic burden. A range of factors can lead to AKI, including ischemia reperfusion (I/R) injury, sepsis, and nephrotoxins (Mehta et al., 2016). Renal I/R is a main cause of AKI and is characterized by a reduction in the tissue blood supply, which causes acute tubular injury. Re-establishing the blood supply following prolonged ischemia stimulates vascular endothelial cells and enhances the generation of reactive oxygen species (ROS). The excessive production of ROS causes oxidative damage in renal tubular epithelial cells, ultimately leading to apoptosis or necrosis (Malek and Nematbakhsh, 2015).

Although the molecular mechanism underlying renal tubular epithelial cell apoptosis in I/R injury is still poorly understood, compelling evidence suggests that mitochondrial dysfunction plays a critical role in apoptosis by producing ROS and releasing cell death factors (Kezic et al., 2016; Yu and Lee, 2016). Cellular stresses, such as ischemia, hypoxia, and glucose deprivation, can cause mitochondrial damage or dysfunction. Therefore, the timely removal of these organelles is critical to cellular homeostasis and viability. Mitophagy, a type of selective autophagy, is a key mechanism in controlling mitochondria quality (Tatsuta and Langer, 2008; Ishihara et al., 2013; Hall and Schuh, 2016). Currently, two main mechanisms of mitophagy have been suggested (Parkin-dependent and Parkin-independent pathways). The Parkin-dependent pathway involves phosphatase and tensin homolog (PTEN)-induced putative kinase 1 (PINK1) and Parkin RBR E3 ubiquitin protein ligase [Parkinson disease 2 (Parkin)], which is the most recognized mitophagy pathway in mammalian cells (Valente et al., 2004). In addition, the Parkin-independent pathway of mitophagy is mediated by Bcl-2 and adenovirus E1B 19-kDa-interacting protein 3 (BNIP3) or BNIP3-like (BNIP3L), which is critical for erythroid maturation (Ashrafi and Schwarz, 2013). Notably, a recent study reported that FUNDC1, a mitochondrial outer-membrane protein, mediates hypoxia-induced mitophagy in mammalian cells and protects the heart from I/R injury (Liu et al., 2012; Zhang et al., 2017).

Augment of liver regeneration (ALR), also known as hepatic stimulator substance, specifically stimulates the regeneration of hepatocytes (LaBrecque and Pesch, 1975). ALR protein is encoded by the growth factor erv1-like (GFER) gene and is highly homologous to the yeast ERV1 family protein (Lange et al., 2001). Accumulating evidence has shown that ALR is expressed ubiquitously in all organs, including the kidney (Tury et al., 2005; Liao et al., 2010). ALR is expressed as a short (15 kDa) and long form (23 kDa). The 15-kDa ALR is mainly secreted from hepatocytes into the extracellular environment, while the 23-kD ALR is reported to in the intermembrane space (IMS) of mitochondria and participates in a redox relay system that mediates the import of proteins to the IMS (Mordas and Tokatlidis, 2015; Nalesnik et al., 2017; Jiang et al., 2019). Increasing evidence has shown that ALR has an antiapoptotic effect (Polimeno et al., 2012). Our earlier study reported that ALR expression is markedly increased in the renal cortex or outer stripe of the outer medulla in rats with AKI (Liao et al., 2009). Intraperitoneal injection of exogenous recombinant ALR enhances the proliferation of renal tubular cells and prevents tubular cell apoptosis (Liao et al., 2010). Furthermore, we found that the knockdown of ALR suppresses mitochondrial biogenesis in I/R injury *in vitro* (Huang et al., 2018). However, if and how ALR participates in the mitophagy pathway of apoptosis in AKI remains unknown, although mitochondria deficiencies are closely associated with apoptosis.

In the present study, we used human kidney proximal tubular (HK-2) cells to establish an I/R model *in vitro* (Huang et al., 2018; Tang et al., 2018), and ALR was knocked down in HK-2 cells by infection with short hairpin RNA (shRNA) lentiviral particles to investigate the effects of ALR on cell apoptosis, mitochondrial dysfunction, and PINK1/Parkin-mediated mitophagy during I/R injury.

## Materials and Methods

### Cells, Reagents, and Antibodies

HK-2 cells were purchased from the American Type Culture Collection (ATCC, Rockville, MD, United States). The ALR shRNA lentiviral and control shRNA lentiviral were purchased from Genechem Co. Ltd (Shanghai, China). The JC-1 fluorescence kit and Annexin V-FITC/PI apoptosis detection kit were obtained from Sigma-Aldrich (MO, United States). MitoSOX was obtained from Invitrogen (Thermo Fisher, MA, United States). 4,6-Diamidino-2-phenylindole was acquired from KeyGen Biotech. Co. Ltd (Nanjing, China). The ALR primer was designed by Sangon Biotech Co., Ltd (Shanghai, China). Total RNA extraction kit and its reverse transcription kit were obtained from Takara Biotechnology (Shiga, Japan). Anti-PINK1, anti-Parkin, anticytochrome c oxidase subunit IV (anti-COX IV), antitranslocase of outer mitochondrial membrane 20 (anti-TOMM20), antitranslocase of inner mitochondrial membrane 23 (anti-TIMM23), anti-P62, and anticleaved caspase-3 (CASP-3) were purchased from Abcam (Cambridge, United Kingdom). Anti-ALR and anti-LC3B were purchased from Thermo Scientific (Thermo Fisher) and Cell Signaling Technology (CST, United States), respectively. Antibeta actin (anti-β-actin) was from Tianjin Sungene Biotech Co., Ltd (Tianjin, China). Cell mitochondrial extraction kits and all secondary antibodies for fluorescent labeling or immunoblot analysis were from Beyotime Biotech Co., Ltd (Shanghai, China).

### Cell Culture and Lentiviral Transduction

HK-2 cells were maintained in Dulbecco’s modified Eagle’s medium (DMEM)/F12 media (Hyclone, Logan, UT, United States) with 10% fetal bovine serum (Hyclone) and 1% penicillin/streptomycin (Invitrogen, Carlsbad, CA, United States). All cells were cultivated at 37°C in a humidified incubator with 5% CO_2_. HK-2 cells were plated in 6-cm culture dishes, then transduced with LV-ALR shRNA and LV-shRNA at a multiplicity of infection (MOI) of 4, according to the manufacturer’s operations. After 72 h, the successfully transduced cells were selected with puromycin (Sigma-Aldrich Corporation). For stable transduction, transduced cells were cultured in complete medium containing 3 μg/ml of puromycin (this concentration of puromycin kills all normal HK-2 cells) for 2 weeks for the next experiment. Western blotting and a quantitative real-time polymerase chain reaction (qPCR) were performed to verify the efficiency of infection. The sequences used to clone the full-length ALR gene were as follows: forward primer, 5′-ATCGGGATCCCGCCACCATGGCGGCGC CCGGCGA-3′; and reverse primer, 5′-CGGGTACCGGTGTCA CAGGAGCCA TCCTTC -3′.

### Model of Ischemia/Reperfusion Injury *in vitro*

To induce I/R injury *in vitro*, HK-2 cells initially were cultured with serum-free media for 12 h to synchronize cell growth. After that, HK-2 cells were washed twice with sterile phosphate-buffered saline (PBS) and incubated in D-glucose free and serum-deprived media in a hypoxic incubator (Thermo Fisher) with 1% O_2_, 5% CO_2_, and 95% N_2_ for 6 h at 37°C. Then, HK-2 cells were moved to a humidified incubator containing 21% O_2_ and 5% CO_2_ and cultured in DMEM/F12 medium supplemented with 10% fetal bovine serum (FBS) for 3, 6, 12, or 24 h. At the end of treatment, cells were monitored morphologically or harvested with the indicated buffers to collect cell lysates for biochemical analyses.

### Lactate Dehydrogenase Assay

A lactate dehydrogenase (LDH) cytotoxicity assay kit (Beyotime Biotech Co., Ltd, Shanghai, China) was used to test cell injury caused by I/R. The observation time point was 12 h of reperfusion followed by 6 h of ischemia. According to the manufacturer’s recommendations, normal and infected lentiviral cells were plated at a density of 3 × 10^4^ per well in 96-well plates. After I/R treatment, the supernatant was collected via porous plate centrifugation (400 × *g* for 5 min). The optical density (OD) at a wavelength of 460 nm was measured using a microplate reader (Molecular Devices, LLC, Sunnyvale, CA, United States). The level of LDH release was calculated according to the instructions. The experiment was performed three times under the same conditions.

### Isolation of Mitochondria

Isolation of the mitochondria was performed with a cell mitochondrion extraction kit (Beyotime Biotech Co., Ltd) in accordance with the manufacturer’s guidelines. In brief, cells were harvested after centrifugation at 500 × *g* for 5 min, then washed with ice-cold PBS. A glass homogenizer was then used to fully homogenize cells after adding the mitochondrial separation reagent and phenylmethylsulfonyl fluoride (PMSF) from the kit. After centrifugation at 1,000 × *g* for 10 min at 4°C, the supernatant was collected, transferred to an ice-cold tube, and centrifuged at 11,000 × *g* for 10 min at 4°C. Subsequently, the pellet was resuspended in mitochondrial lysate containing PMSF and contained the mitochondrial proteins.

### Confocal Microscopy

Confocal microscopy was used to investigate whether LC3, PINK1, or Parkin colocalized with the mitochondria. TOMM20, a mitochondrial outer membranous protein, was used to label mitochondria. HK-2 cells were seeded onto a glass-bottomed dish (NEST, Beijing, China) for confocal microscopic observation. After I/R treatment, HK-2 cells were rinsed twice with PBS and then fixed with 4% paraformaldehyde for 20 min at 37°C. All cells were permeabilized with 0.3% Trion X-100 for 20 min and washed with PBS. Next, the cells were blocked with 5% bovine serum albumin (BSA) and incubated overnight at 4°C in primary antibody (concentration of antibodies according to manufacturer’s instruction). The cells were rinsed twice with PBS and then further incubated with fluorescein isothiocyanate (FITC)- or tetramethylrhodamine isothiocyanate (TRITC)-conjugated secondary antibody at room temperature for 1 h. At last, 4,6-diamidino-2-phenylindole was used to dye the nuclei. The images were detected and analyzed via laser scanning confocal microscopy (Nikon, Ti2000, Japan).

### Quantitative Real-Time Polymerase Chain Reaction

Total RNA was extracted from lentiviral infected and normal HK-2 cells using total RNA extraction kits (Takara, Kusatsu, Japan). Total RNA was reverse-transcribed to cDNA using a PrimeScript^TM^ II Reverse Transcriptase kit (Takara). Real-time PCR was performed using SYBR Premix (Takara). For quantitative analysis, the ALR gene and glyceraldehyde 3-phosphate dehydrogenase (GAPDH) as a positive control were amplified by SYBR Premix using a CFX Connect Real-Time system (Bio-Rad, Hercules, CA, United States). Relative gene expression was then analyzed using 2^–ΔΔCt^. The human ALR and GAPDH primers were as follows, ALR: forward, 5′-CAG AAG CGG GAC ACC AAG TTT-3′; reverse, 5′-CAC ACT CCT CAC AGG GGTAA-3′. GAPDH: forward,5′-TGA CTT CAA CAG CGA CAC CCA-3′; reverse, 5′-CAC CCT GTT GCT GTA GCC AAA-3′. Three independent experiments were repeated.

### Western Blotting

Cells were lysed in radioimmunoprecipitation assay (RIPA) buffer (Bioteke Corporation, Wuxi, China) containing protease and phosphatase inhibitors (Bioteke Corporation); then, protein lysates were prepared from cell homogenate via centrifugation according to the instructions. Protein samples were separated by 10 or 15% sodium dodecyl sulfate (SDS)-polyacrylamide gel electrophoresis and transferred to polyvinylidene difluoride membranes (EMD Millipore, Bedford, MA, United States). The membranes were blocked with 5% BSA in TBST for at least 1 h and then incubated in specific primary antibodies at 4°C for at least 12 h. Subsequently, membranes were soaked in TBST containing secondary antibodies for 1 h and visualized by enhanced chemiluminescence detection. The gray values of the target bands were analyzed using the ImageJ software (National Institutes of Health, Bethesda, MD, United States).

### Transmission Electron Microscopy

After I/R treatment, HK-2 cells from each group were collected and then fixed with 2.5% glutaraldehyde and embedded by epoxy resin. The sample slice thickness was ∼50 nm. The morphologic alterations of mitochondria were detected by TEM (Hitachi, Tokyo, Japan).

### Detection of Mitochondrial Membrane Potential

Mitochondrial membrane potential (MMP) was assessed using a JC-1 fluorescence dye. Normal HK-2 cells and I/R injured HK-2 cells were rinsed twice with PBS and incubated with JC-1 (300 nM) for 20 min at 37°C. Cells were washed twice with JC-1 staining buffer (1×), then incubated with complete medium for detection using a confocal microscope, or collected for measurement via flow cytometry with 485 nm excitation and 528 nm emission wavelengths.

### Detection of Intracellular ATP

The intracellular ATP levels of HK-2 cells was assessed using an ATP assay kit (Beyotime Biotech, Shanghai, China), Experimental methods and procedures were operated according to the manufacturer’s protocol. The ATP content of the cells was measured in nmol/mg protein.

### Detection of Mitochondrial ROS

Normal HK-2 cells and I/R injured HK-2 cells were washed twice with PBS. Then, HK-2 cells were incubated in 5 μM MitoSOX for 10 min in the dark at 37°C. Cells in each group were collected separately, and the fluorescence levels of MitoSOX of each group was measured by flow cytometry with 488 nm excitation and 525 nm emission wavelengths.

### Measurement of Apoptosis

Apoptosis was measured by immunoblot analysis of cleaved caspase-3 and flow cytometry. The process of immunoblot analysis of cleaved CASP-3 was described under Western blotting. Flow cytometry was carried out by double-labeling of Annexin V and propidine iodide following the manufacturer’s instructions. Three independent experiments were repeated.

### Statistical Analyses

All data are represented as mean ± standard deviation (SD). Statistical analysis between two groups was performed with Student’s *t* test. Statistical analysis between multiple groups was performed with one-way ANOVA followed by Tukey’s *post hoc* tests. Significant difference was considered as *p* < 0.05.

## Results

### Mitochondrial Damage, Mitophagy, and Activation of ALR Induced in HK-2 Cells by I/R

**FIGURE 1:**
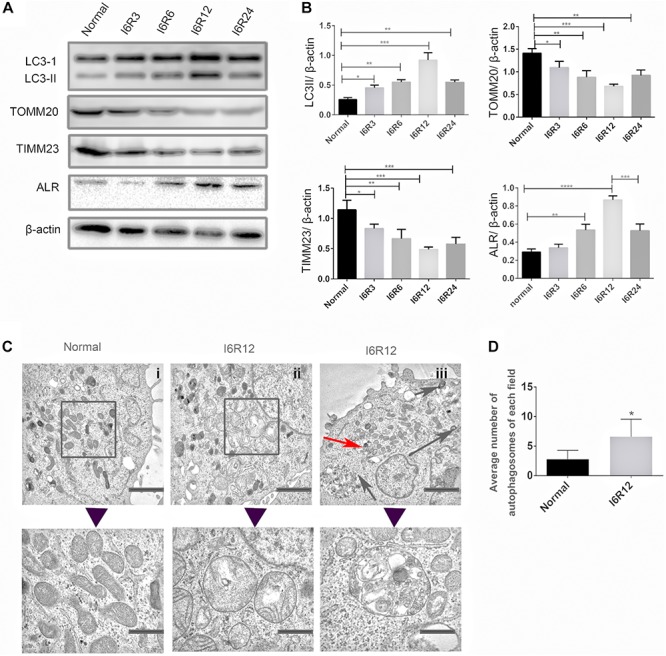
Mitochondrial damage, mitophagy, and activation of augmenter of liver regeneration (ALR) were induced in HK-2 cells by ischemia–reperfusion (I/R). **(A,B)** Immunoblot analysis and quantification of ALR, LC3, translocase of outer mitochondrial membrane 20 (TOMM20), and translocase of inner mitochondrial membrane 23 (TIMM23). **(C,D)** Representative TEM images of mitochondrial morphology (square box), mitophagosomes (red arrow), and autophagosomes (black arrow) in HK-2 cells. The observation timepoint was I6R12. Data are shown as a bar graph of the number of autophagosomes from five images of each group. Scale bar: 2 μm. The images in the lower row show the amplification of the normal mitochondria, damaged mitochondria, and mitophagosomes. Scale bar: 200 nm. Data represent means ± SD. *n* = 3. *p < 0.05, **p < 0.01, ***p < 0.001, ****p < 0.0001.

In the present study, immunoblot analysis showed that the protein level of LC3-II, an autophagy biomarker, was significantly upregulated after reperfusion, peaking at 12 h, followed by a decrease. The change in LC3-II was associated with a gradual reduction in the mitochondrial inner membrane protein TIMM23 and translocase of outer mitochondrial membrane 20 (TOMM20). These results indicated that mitochondria clearance or mitophagy was induced in HK-2 cells subjected to I/R and was more notable after 6 h of ischemia and 12 h of reperfusion (I6R12; Figures 1A,B). Thus, I6R12 was selected as an observation point to perform the next experiments. Then, we used TEM to examine the changes of mitochondrial morphology and mitophagosome in HK-2 cells. After I/R treatment, mitochondrial swelling, loss of mitochondrial cristae, and vacuole formation in the mitochondria matrix were apparent in HK-2 cells (Figure 1C-ii), and mitophagosomes, as well as autophagosomes, were also found (Figure 1C-iii). There were more autophagosomes in the I/R group (Figure 1D). These findings further confirmed the activation of mitochondrial damage and mitophagy were induced by I/R.

Because 23-kD ALR is mainly localized in the IMS of mitochondria, and our previous study showed that 23-kD ALR had a protective role in mitochondria via reduction in ROS (Xia et al., 2015). We investigated whether 23-kD ALR was involved in I/R-induced mitophagy. We found that 23-kD ALR protein expression gradually increased at 12 h and decreased after 24 h. These changes in 23-kD ALR protein expression indicated that 23-kD ALR might be involved in the process of mitophagy induced by I/R.

### PINK1/Parkin Pathway Was Activated by I/R in HK-2 Cells

**FIGURE 2:**
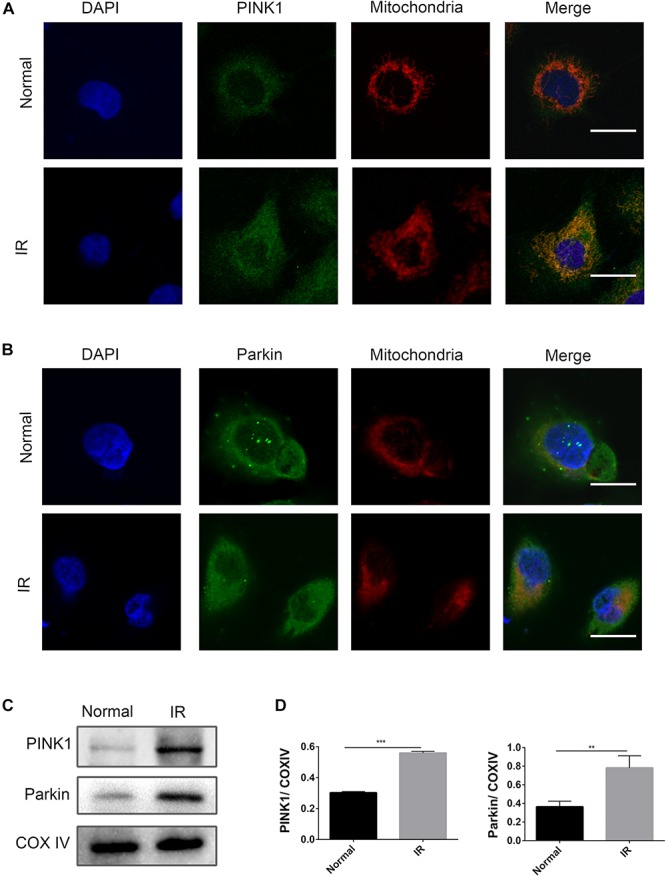
PINK1/Parkin pathway was activated by ischemia–reperfusion (I/R) in HK-2 cells. (**A**,**B**) Representative images of immunofluorescence double-labeling PINK1/Parkin and translocase of outer mitochondrial membrane 20 (TOMM20). TOMM20 was used as a mitochondrial tracker. PINK1 or Parkin was labeled by fluorescein isothiocyanate (FITC) (green), TOMM20 was labeled by tetramethylrhodamine isothiocyanate (TRITC) (red). Strong colocalization signals in I/R group were seen as yellow dots after merging, suggesting that PINK1 or Parkin gathered on the mitochondria. Scale bar = 50 μm. **(C,D)** Immunoblot analysis and quantification of PINK1 and Parkin. Data represent means ± SD. *n* = 3. **p < 0.01, ***p < 0.001.

In kidney disease, the PINK1/Parkin pathway was reported to play a dominant role in mitophagy (Lin et al., 2019). In this study, an activation of the PINK1/Parkin pathway at the time point of I6R12 was measured using immunofluorescence and immunoblot analysis. Immunofluorescence showed many clear green dots as a sign of PINK1 or Parkin in HK-2 cells after I/R. In contrast, only weak green immunofluorescence was observed in the normal group. Costaining with TOMM20, a mitochondrial marker, showed colocalization of PINK1/Parkin with mitochondria in I/R-treated HK-2 cells, indicating the activation of the PINK1/Parkin pathway induced by I/R. Consistent with immunofluorescence, immunoblot analysis showed that PINK1/Parkin from mitochondria was upregulated by I/R (Figure 2).

### Silencing of ALR Increases HK-2 Cells Susceptibility to I/R Treatment

**FIGURE 3:**
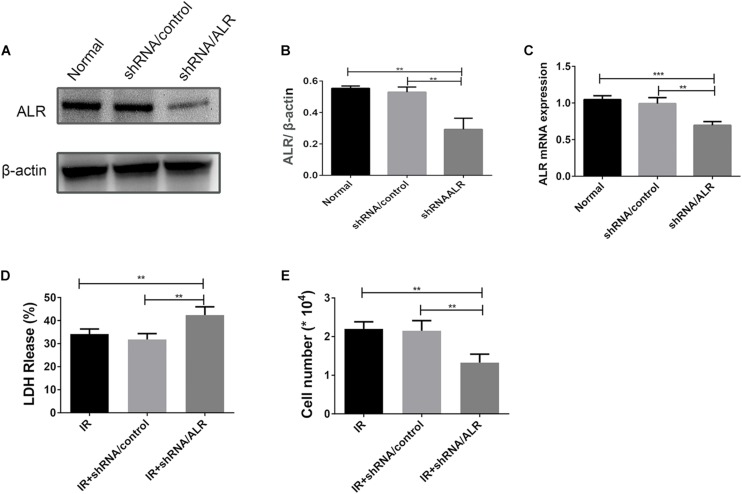
Silencing of augmenter of liver regeneration (ALR) increases HK-2 cells Susceptibility to ischemia–reperfusion (I/R) treatment. **(A,B)** Immunoblot analysis and quantification of ALR. **(C)** The expression of ALR messenger RNA (mRNA) was measured via quantitative real-time PCR (qPCR). Data represent means ± SD. *n* = 3. **p < 0.01, ***p < 0.001. **(D)** The lactate dehydrogenase (LDH) released in the supernatant of cultured cells was measured at the indicated time. A significant increase was observed in the short hairpin (shRNA)/ALR group when compared with the shRNA/control group or I/R group. **(E)** The cell number was counted in three groups at I6R12. The cell number of shRNA/ALR group clearly decreased after I/R. Data represent means ± SD. *n* = 4. **p < 0.01.

HK-2 cells were transduced with a lentiviral vector carrying ALR shRNA to knock down the expression of ALR. The expression of ALR was examined by qPCR and immunoblotting analysis. The results of immunoblotting analysis showed that the ALR protein expression in the shRNA/ALR group was significantly lower than the normal or shRNA/control group (0.29 ± 0.07, 0.55 ± 0.01, 0.53 ± 0.03, *p* < 0.01; Figures 3A,B). Similar results were obtained by qPCR. These results demonstrate the transduction efficiency of HK-2 cells. LDH is a stable cytoplasmic enzyme in all cells and is released rapidly following plasma membrane damage and thus used as an indicator of cell injury (Abdel-Latif et al., 2006). To investigate whether ALR has a protective effect for HK-2 cells in I/R treatment, we determined the levels of LDH release in all three groups. The results showed that the release of LDH in the shRNA/ALR group was clearly increased at the timepoint of I6R12 (Figure 3D). In addition, the number of cells in the shRNA/ALR group was significantly decreased after I/R compared with the shRNA/control or I/R group (1.33 × 10^4^ ± 0.22, 2.15 × 10^4^ ± 0.26, 2.2 × 10^4^ ± 0.18, *p* < 0.01; Figure 3E). These results indicated that knockdown of ALR enhanced I/R injury in HK-2 cells.

### Silencing of ALR Inhibited I/R-Induced Mitophagy in HK-2 Cells

**FIGURE 4:**
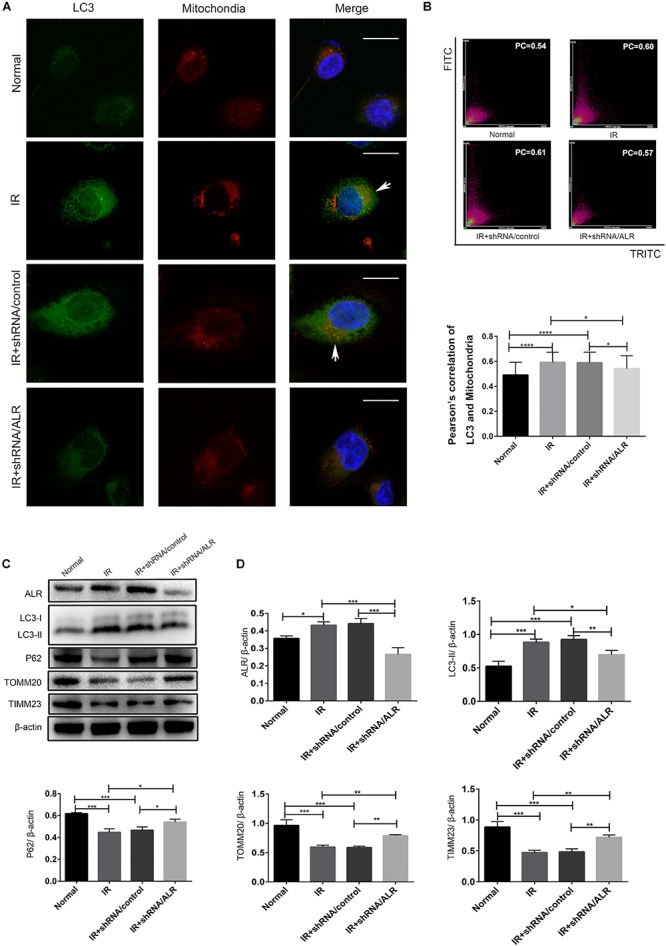
Silencing of augmenter of liver regeneration (ALR) inhibited ischemia–reperfusion (I/R)-induced mitophagy in HK-2 cells. **(A,B)** Representative images and quantification of immunofluorescence double-labeling LC3B and translocase of outer mitochondrial membrane 20 (TOMM20) in different groups. TOMM20-labeled mitochondria (red) and LC3-labeled autophagosome (green). Scale bar: 50 μm. the Pearson’s correlation columns were shown in bar graph format. Sixty cells from three independent experiments were analyzed. **(C,D)** Immunoblot analysis and quantification of ALR, LC3, P62, TOMM20, and translocase of inner mitochondrial membrane 23 (TIMM23). Data represent the means ± SD. *n* = 3. *p < 0.05, **p < 0.01, ***p < 0.001, ****p < 0.0001.

To investigate whether ALR protects HK-2 cells from I/R injury by regulating mitophagy, we examined the occurrence of mitophagy in HK-2 cells. Confocal microscopy revealed that the shRNA/ALR group had very few green puncta of the autophagosome marker, LC3, but a large number of green puncta was shown in the shRNA/control group. Costaining with mitochondria revealed a reduction in colocalized autophagosomes and mitochondria in the shRNA/ALR group based on the Pearson’s correlation coefficient to evaluate the overlap of LC3 and TOMM20. These results showed that the formation of mitophagosomes was reduced after silencing ALR (Figures 4A,B). Consistent with immunofluorescence, immunoblot analysis of P62, LC3-II, TIMM23, and TOMM20 revealed decreased autophagy activation and mitochondrial clearance (Figures 4C,D).

### Silencing of ALR Inhibited the Activation of the PINK1/Parkin Pathway in HK-2 Cells During I/R

**FIGURE 5:**
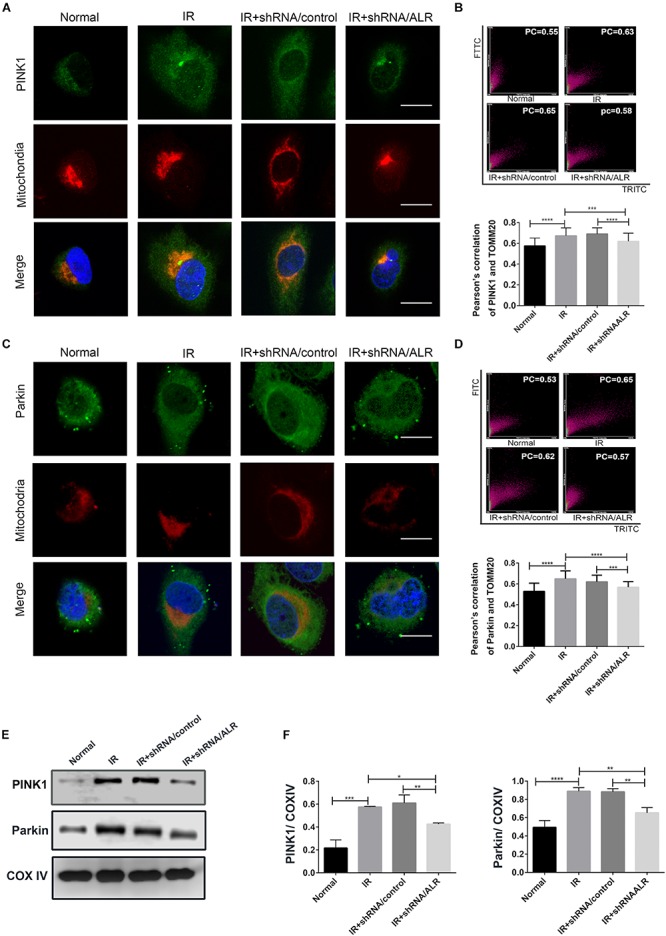
Silencing of augmenter of liver regeneration (ALR) inhibited the activation of PINK1/Parkin pathway in HK-2 cells during ischemia–reperfusion (I/R). **(A–D)** Representative images and quantification of immunofluorescence double-labeling PINK1/Parkin and mitochondrial marker [translocase of outer mitochondrial membrane 20 (TOMM20)]. PINK1 or Parkin was labeled with fluorescein isothiocyanate (FITC) (green), mitochondria was labeled with tetramethylrhodamine isothiocyanate (TRITC) (red). Scale bar = 50 μm. the Pearson’s correlation columns were shown in bar graph format. Sixty cells from three independent experiments were analyzed. **(E,F)** Immunoblot analysis and quantification of PINK1 and Parkin. Data represent means ± SD. *n* = 3. *p < 0.05, **p < 0.01, ***p < 0.001, ****p < 0.0001.

Our previous results showed that silencing of ALR inhibited I/R-induced mitophagy in our experimental model. We also found that PINK1 and Parkin was activated by I/R, which could be critical regulatory factors in mitophagy control. Therefore, we further investigated the possibility that ALR might mediate I/R-induced mitophagy via the PINK1/Parkin pathway *in vitro*. The results showed that, in response to I/R, HK-2 cells with ALR shRNA had a decrease in PINK1 and Parkin protein expression, as well as colocalization with mitochondria in immunofluorescence staining compared with cells with control shRNA (Figure 5). Consequently, the signaling pathway of ALR regulating mitophagy may involve PINK1 and Parkin.

### Silencing of ALR Aggravated I/R-Induced Mitochondrial Dysfunction in HK-2 Cells

**FIGURE 6:**
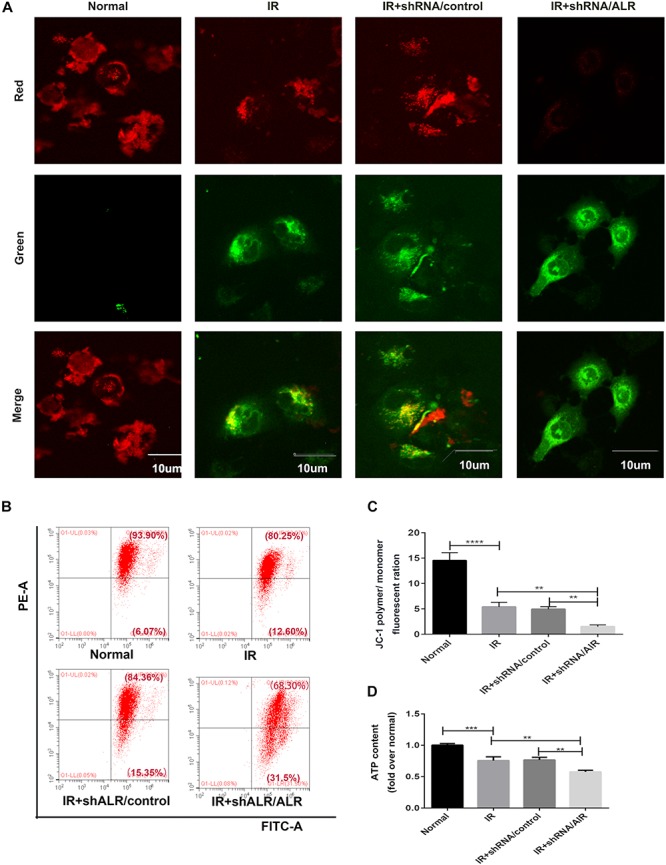
Silencing of augmenter of liver regeneration (ALR) aggravated ischemia–reperfusion (I/R)-induced mitochondrial dysfunction in HK-2 cells. **(A)** Representative fluorescence images of HK-2 cells stained by JC-1. The picture was detected by confocal laser scanning microscopy (CLSM). Scale bar = 10 μm. **(B,C)** Representative images and analysis of mitochondrial membrane potential (MMP) via flow cytometry. **(D)** ATP contents. Using an ATP assay kit to measure ATP levels for each group. Data represent as the means ± SD. *n* = 3. **p < 0.01, ***p < 0.001, ****p < 0.0001.

Because ALR may regulate mitophagy, we further investigated the effects of ALR on I/R-induced mitochondrial dysfunction in HK-2 cells. As shown in Figures 6A–C, a loss of mitochondrial membrane potential was noted under I/R treatment, which was assessed by JC-1 staining and flow cytometry, and was further accelerated by the knockdown of ALR. In addition, silencing of ALR aggravated the inhibitory effect of I/R on ATP production (Figure 6D).

### Silencing of ALR Enhanced I/R-Induced Mitochondrial ROS Generation

**FIGURE 7:**
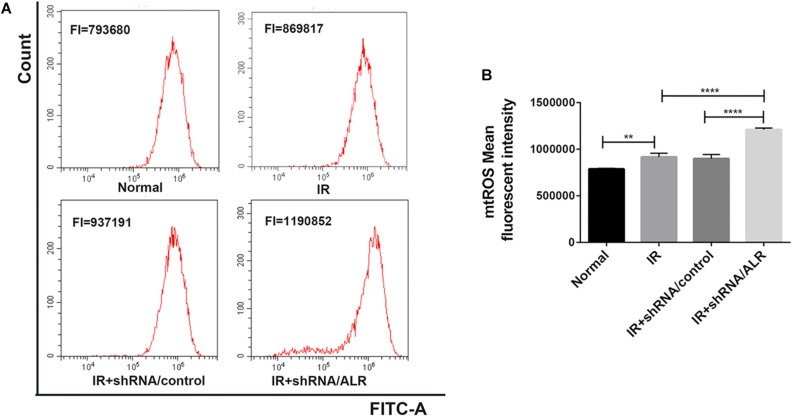
Silencing of augmenter of liver regeneration (ALR) enhanced ischemia–reperfusion (I/R)-induced mitochondrial reactive oxygen species (ROS) generation. **(A)** The levels of mitochondrial ROS were determined by flow cytometry. **(B)** Quantitation of MitoSOX were shown in a bar graph. Data represent means ± SD. *n* = 3. **p < 0.01, ****p < 0.0001.

Mitochondrial dysfunction induces harm via the induction of mitochondrial ROS production or mitochondrial permeability transition, or both. The knockdown of ALR aggravated mitochondrial dysfunction. Subsequently, we explored the effect of silencing the *ALR* gene on I/R-induced mitochondrial ROS generation; we used MitoSOX kit to measure the production of mitochondrial ROS by flow cytometry. The results showed that mitochondrial ROS levels were significantly increased in the shRNA/ALR group after I/R, compared with shRNA/control group (1.209 × 10^6^ ± 17,738, 0.898 × 10^6^ ± 45,752, *p* < 0.0001; Figure 7).

### Silencing of ALR-Sensitized HK-2 Cells to I/R-Induced Apoptosis

**FIGURE 8:**
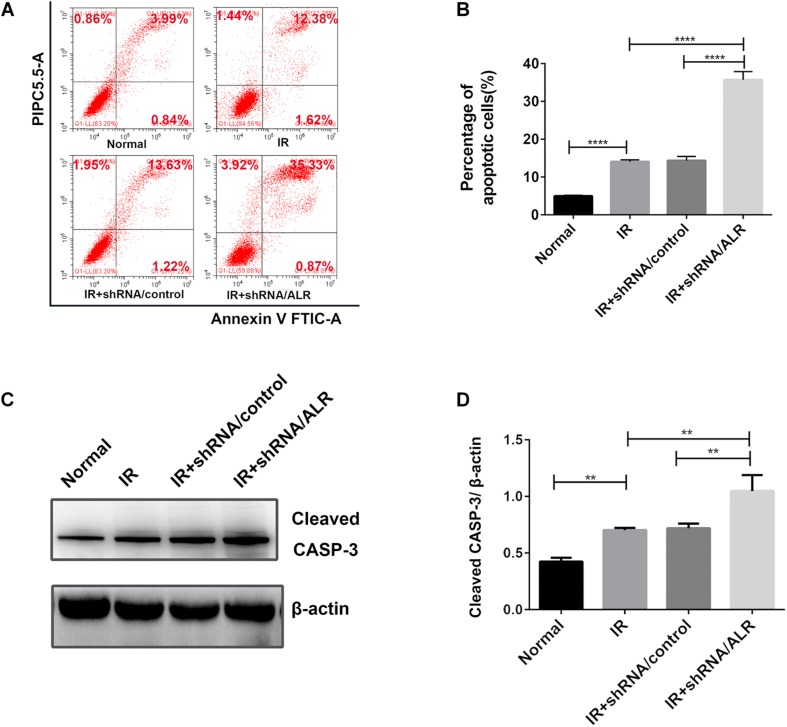
Silencing of augmenter of liver regeneration (ALR)-sensitized HK-2 cells to induced apoptosis. Apoptosis was determined by flow cytometry and activated caspase-3 (CASP-3). **(A,B)** Representative plot and quantitation analysis of apoptosis measured by flow cytometry. **(C,D)** Immunoblot analysis and quantification of activated CASP-3. Data represent means ± SD. *n* = 3. **p < 0.01, ****p < 0.0001.

To determine the effect of silencing of the *ALR* gene on I/R-induced renal tubular cell injury, we initially investigated I/R-induced apoptosis of HK-2 cells via flow cytometry. Cell apoptosis in the shALR/ALR group was higher than that of shALR/control group (35.71 ± 2.157%, 14.35 ± 1.105%, *p* < 0.0001; Figures 8A,B). The flow cytometer assay results were confirmed by Western blot analysis of activated CASP-3. In response to I/R, the level of activated CASP-3 in HK-2 cells with silencing *ALR* was significantly higher than that of the short hairpin RNA (shRNA)/control (1.05 ± 0.14, 0.72 ± 0.05, *p* < 0.01; Figures 8C,D). Collectively, these findings suggested that ALR played a cytoprotective role in I/R injury *in vitro*.

## Discussion

The main pathological phenotype of AKI is tubular damage, including apoptosis. ALR has been reported to have an antiapoptosis function (Polimeno et al., 2012); however, the mechanism underlying this effect remains unclear. In this study, we first showed that activation of ALR and mitochondrial dysfunction were induced in I/R-AKI *in vitro*. Then, we showed that a deficiency in ALR increased mitochondrial ROS production and mitochondrial deficiencies accompanied with inhibition of PINK1/Parkin-mediated mitophagy, contributing to cell apoptosis in I/R *in vitro*. Therefore, we proposed that ALR may protect renal tubular epithelial cells from I/R injury by promoting Pink1/Parkin-mediated mitophagy.

Mitochondria are the center of energy metabolism in the cell and believed to be the primary subcellular target of I/R injury. Evidence of mitochondrial dysfunction involved in human AKI has been reported in an electron microscopy study of kidney specimens from patients who had died from septic shock (Emma et al., 2016). Pathological alterations in mitochondrial structure and function have been observed in renal tubular cells in various experimental models of AKI (Parikh et al., 2015; Kezic et al., 2016; Yu and Lee, 2016). In this study, we established an *in vitro* I/R model with glucose-free culture medium and hypoxia reoxygenation according to previous studies (Jiang et al., 2010; Dong et al., 2015; Zhang et al., 2015; Huang et al., 2018). The changes in LC3-II during I/R was associated with a marked reduction in TIMM23 and TOMM20, indicating activation of mitochondrial clearance. TEM also showed damaged morphological changes in mitochondria in HK-2 cells during I/R. Aggravation of mitochondrial dysfunction was shown in HK-2 cells, as evidenced by a decrease in ATP production and mitochondrial membrane potential, which led to an increase in mitochondrial ROS and cell apoptosis. These results suggested that mitochondria damage or mitochondrial dysfunction is involved in the pathogenesis of I/R-AKI *in vitro* and closely associated with cell injury. Our previous study reported that ALR has an antiapoptotic effect in I/R injury (Liao et al., 2010). In this I/R model, we explored the link between ALR and I/R-induced mitochondrial dysfunction. In the process of I/R, ALR expression was also significantly upregulated with a time-dependent pattern, peaking at 12 h. Knockdown of ALR aggravated I/R-induced mitochondrial dysfunction, mitochondrial ROS, and cell apoptosis compared with the shRNA/control group. These observations indicated that ALR may take part in the process of I/R-induced mitochondrial dysfunction and protect cells from I/R injury. However, the underlying mechanism needs further study.

Mitophagy plays an important role in the clearance of damaged mitochondria (Linkermann et al., 2014). The reduction of mitophagy significantly exacerbated cell apoptosis and tissue damage of AKI (Zhao et al., 2017; Tang et al., 2018; Yang et al., 2018). In the present study, activation of mitophagy was found in HK-2 cells after I/R treatment. After silencing ALR, a reduction in mitophagy accompanied with an increase in mitochondrial dysfunction, mitochondria ROS, and cell apoptosis also occurred during I/R. Our research confirmed that mitophagy has a protective effect in AKI. Accordingly, ALR may play a protective role in I/R injury and mitochondria function via promoting mitophagy. It is noteworthy that the interaction between mitophagy and cell death is complex (Marino et al., 2014); emerging evidence also suggests that mitophagy is a possible mechanism for cell death programs in chronic obstructive pulmonary disease (COPD) (Mizumura et al., 2014). Therefore, the effect of mitophagy in different pathological conditions needs further investigation.

Many studies have reported that PINK1/Parkin signaling controls the selective degradation of dysfunctional mitochondria by mitophagy in acute kidney disease (Zhao et al., 2017; Tang et al., 2018; Lin et al., 2019). In healthy mitochondria, PINK1 is constitutively inserted into the mitochondria inner membrane and rapidly degraded by proteolysis to keep a low level of expression. When mitochondria are damaged, PINK1 accumulates on the mitochondrial outer membrane (MOM), which then recruits Parkin from the cytosol and phosphorylates its ubiquitin ligase. After phosphorylation, Parkin ubiquitinates various proteins in the MOM and delivers ubiquitin mitochondria to autophagosomes via combing autophagy protein P62 and microtubule-associated proteins 1 light chain 3 beta (MAP1LC3B/LC3B), inducing mitophagy and removal of damaged mitochondria (Geisler et al., 2010). In this study, we first verified the PINK1/Parkin pathway in I/R injury. Next, we found that silencing of ALR suppressed the activation of PINK1/Parkin pathway via double immunofluorescent staining and Western blot. These results suggested that ALR may regulate mitophagy via the PINK1/Parkin pathway. Furthermore, it seems that multiple molecules, such as BNIP3 and FUNDC1, are involved in mitophagy. Additional studies are needed to elucidate the exact mechanisms through which ALR exerts its regulatory function on mitophagy.

Interestingly, mounting evidence has suggested that P62 may be crucial for PINK1/Parkin-mediated mitophagy (Geisler et al., 2010). However, another study found that mitochondrial removal has taken place independently of P62 (Narendra et al., 2010). A recent study showed that instead of P62, optineurin was recruited to damaged mitochondria and transferred damaged mitochondrial to the autophagosome in PINK1/Parkin-mediated mitophagy via its LC3 interaction region domain (Wong and Holzbaur, 2014). In the present study, our results provided support that P62 may be essential in PINK1/Parkin-induced mitophagy, while the expression of P62 changed with LC3, which was consistent with the level of autophagy. Hence, the exact processes of PINK1/Parkin-mediated mitophagy need further investigation.

**FIGURE 9:**
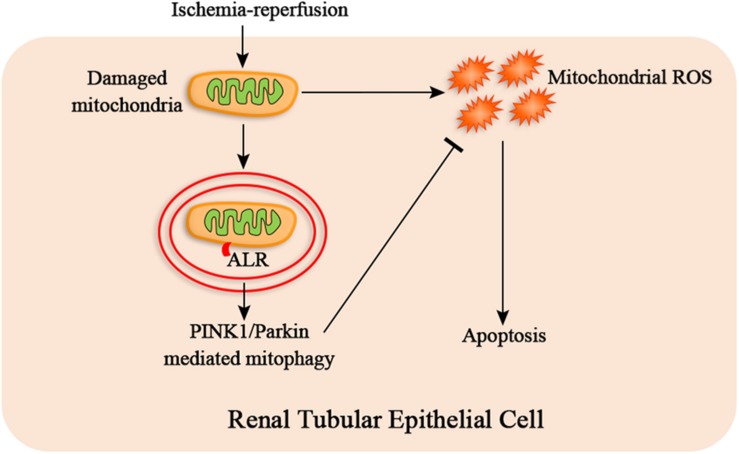
The hypothetical mechanism of protective effect of augmenter of liver regeneration (ALR) in ischemia–reperfusion (I/R)–acute kidney injury (AKI) *in vitro*. I/R causes the mitochondrial damage of renal tubular epithelial cells, which induces mitochondrial reactive oxygen species (ROS) and apoptosis. ALR may protect renal tubular epithelial cells from apoptosis via PINK1/Parkin-mediated mitophagy, which remove the damaged mitochondria and reduce mitochondrial ROS production.

In summary, our research provides evidence that ALR attenuates I/R-induced mitochondrial pathway of apoptosis by reducing mitochondrial ROS production and mitochondrial dysfunction. The mechanism of protective effect of ALR may be closely associated with promoting PINK1/Parkin-mediate mitophagy (Figure 9). Consequently, ALR may serve as a new therapeutic target for AKI in the future.

## Data Availability Statement

All datasets generated for this study are included in the article/supplementary material.

## Ethics Statement

This experiment is an *in vitro* experiment without any ethics problems.

## Author Contributions

D-JZ performed the experiments and wrote the manuscript. HS performed, measured, and analyzed the confocal laser-scanning microscopy experiments. X-HL and W-QH revised and corrected the manuscript. LZ and QL designed the study and helped in analyzing and interpretation of data. All authors read and approved the manuscript.

## Conflict of Interest

The authors declare that the research was conducted in the absence of any commercial or financial relationships that could be construed as a potential conflict of interest.
